# P-1134. Use of Respiratory Panel Polymerase Chain Reaction (RPP): Challenging Diagnostic Stewardship

**DOI:** 10.1093/ofid/ofae631.1321

**Published:** 2025-01-29

**Authors:** Marisol Fernandez, Rachel Downey, Alec Wesolowski

**Affiliations:** Dell Children's Medical Center of Central Texas, Dell Medical School at UT Austin, Austin, Texas; Dell Children's Medical Center of Central Texas, Austin, Texas; Dell Children's Medical Center, Austin, Texas

## Abstract

**Background:**

National diagnostic stewardship initiatives recommend against routine use of broad respiratory pathogen panels (RPP) for children with suspected respiratory viral illness. Use of RPP testing increased at our institution after 2020, likely related to the ongoing pandemic. Our antimicrobial stewardship team sought to meet in person with ordering services to improve diagnostic stewardship, reserving RPP for cases in which broad testing was likely to affect treatment. The aim of our study was to assess if the handshake stewardship initiative was enough to decrease the number of RPP ordered. A secondary aim was to evaluate the proportion of positive RPP tests that resulted in treatment change before and after the stewardship initiative.

**Figure:**
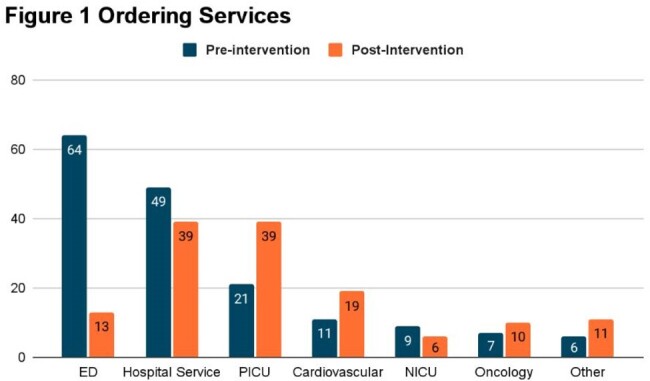

**Methods:**

This single-center, retrospective study included pediatric patients with RPP test in January to February 2023 (pre-intervention) and January to February 2024 (post-intervention). Stewardship interventions took place in June 2023. Of the ordering services included, only the emergency department (ED) and hospital service opted to meet in person. Cases were compared pre and post intervention for length of stay, race, underlying medical conditions, results of RPP, ordering service, and documented change in treatment plan based on the test results.

**Figure:**
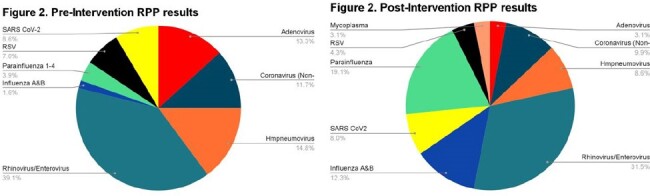

**Results:**

172 and 290 RPP orders in the pre and post intervention groups were included. 101 (59%) tests were positive in the pre-intervention group compared to 136 (47%) in the post-intervention. Figure 1 shows the distribution of the ordering medical services. There was a significant decrease in RPP orders in the ED post intervention (p < 0.001) (fig 1). Among patients with a positive RPP there was a treatment change in 20 (20%) pre vs 36 (26%) post-intervention (p=0.298). Figure 2 shows distribution of pathogens among positive testing. The most common viral pathogen identified in both groups was rhinovirus/enterovirus.

**Conclusion:**

In-person handshake stewardship has the potential to guide appropriate utilization of diagnostic tests. RPP testing did not result in changes in treatment for the majority of patients regardless of handshake stewardship guidance. Future work will include developing a diagnostic algorithm to refine selection of patients to be tested.

**Disclosures:**

**All Authors**: No reported disclosures

